# C-reactive protein for predicting all-cause mortality in patients with acute ischemic stroke: a meta-analysis

**DOI:** 10.1042/BSR20181135

**Published:** 2019-02-19

**Authors:** Bo Yu, Ping Yang, Xuebi Xu, Lufei Shao

**Affiliations:** 1 Department of Neurology, Qilu Hospital of Shandong University, Jinan 250012, Shandong, China; 2 Department of Neurology, General Hospital of Ningxia Medical University, Yinchuan 750004, Ningxia, China

**Keywords:** acute ischemic stroke, all-cause mortality, C-reactive protein, meta-analysis

## Abstract

Studies on the association of C-reactive protein (CRP) with all-cause mortality in acute ischemic stroke patients have yielded conflicting results. The objective of this meta-analysis was to evaluate the prognostic value of CRP elevation in predicting all-cause mortality amongst patients with acute ischemic stroke. We searched the original observational studies that evaluated the association of CRP elevation with all-cause mortality in patients with acute ischemic stroke using PubMed and Embase databases until 20 January 2018. Pooled multivariate-adjusted hazard ratio (HR) with 95% confidence intervals (CI) of all-cause mortality was obtained for the highest compared with the lowest CRP level or per unit increment CRP level. A total of 3604 patients with acute ischemic stroke from eight studies were identified. Acute ischemic stroke patients with the highest CRP level were independently associated with an increased risk of all-cause mortality (HR: 2.07; 95% CI: 1.60–2.68) compared with the lowest CRP category. The pooled HR of all-cause mortality was 2.40 (95% CI: 1.10–5.21) for per unit increase in log-transformed CRP. Elevated circulating CRP level is associated with the increased risk of all-cause mortality in acute ischemic stroke patients. This meta-analysis supports the routine use of CRP for the death risk stratification in such patients.

## Introduction

Stroke remains the second most common cause of death and the leading cause of disability worldwide [1]. Ischemic stroke is the most common type of stroke, comprising 87% of all types of stroke [2]. Ischemic stroke causing permanent disability is associated with a remarkable economic and social burden [3]. A decreasing trend in total mortality of ischemic stroke has been observed in many regions [4–6]. The markedly increased number of ischemic stroke survivors may be partly attributed to improvements in in-hospital management [7]. Despite the decline in overall ischemic stroke hospitalizations in the United States, the age-specific acute ischemic stroke hospitalization rates increased for patients aged 25–64 years [8]. Therefore, early prediction of outcomes after acute ischemic stroke is clinically important for optimized care.

Inflammation plays an important role in the pathophysiology of atherosclerosis and ischemic stroke [9,10]. Several inflammatory cytokines have been identified as cardiovascular and functional outcome predictors after ischemic stroke [11]. C-reactive protein (CRP) is a frequently studied inflammatory biomarker that is implicated in all stages of ischemic stroke [12]. Elevated CRP level is independently associated with the excessive risk of ischemic stroke [13]. However, studies on the association of CRP elevation and all-cause mortality risk in patients with acute ischemic stroke have yielded inconsistent results [14,15]. Previous meta-analysis only evaluated the association between CRP elevation and poor functional outcome but did not focus on the all-cause mortality outcome in patients with ischemic stroke [16]. To date, the prognostic value of CRP elevation in patients with acute ischemic stroke who have a high risk of death is still poorly studied.

Currently, no previous meta-analysis has quantitatively examined the association between CRP and all-cause mortality in patients with acute ischemic stroke. We aimed to evaluate the association of CRP elevation with all-cause mortality risk in patients with acute ischemic stroke by conducting a meta-analysis on available observational studies.

## Materials and methods

### Data source and literature search

This meta-analysis followed the reporting checklist of the Meta-analysis of Observational Studies in Epidemiology [17]. Two authors independently searched PubMed and Embase databases from their inception to 28 February 2018. Search terms included ‘C-reactive protein’ or ‘CRP’ and ‘mortality’ or ‘death’ or ‘survival’ and ‘stroke’ or ‘cerebral infarction’ or ‘cerebrovascular accident’. In addition, reference lists of all relevant articles were also manually checked to identify additional studies. Language was restricted to English.

### Study selection

Studies were eligible if the following conditions were met: (i) enrolled adult patients with acute ischemic stroke; (ii) determined baseline CRP levels after the symptom onset of ischemic stroke or on admission; (iii) provided data on all-cause mortality; (iv) at least 3 months of follow-up duration; and (v) reported multivariable adjusted risk estimate of all-cause mortality associated with the CRP elevation or continuous value of CRP level. Studies were excluded if: (i) included hemorrhagic stroke patients; (ii) unadjusted risk estimate reported; (iii) functional outcome as the outcome of prognosis; and (iv) duplicated publication, conference abstracts, or letters.

### Data extraction and quality assessment

The following data were abstracted from each of the eligible studies: first author, publication year, country of origin, study type, sample size, gender, median or mean age of patients, CRP cut-off value, follow-up duration, number of deaths, fully adjusted risk estimate, and adjustment for covariates. The methodological quality of each article was assessed using the Newcastle–Ottawa Scale (NOS) [18] and study achieving 7 or over points was regarded high quality. Any discrepancy between two authors was settled by discussion.

### Data analysis

All statistical analyses were carried out with STATA (version 12.0, Stata, College Station, TX, U.S.A.). Summary risk ratio [RR) with 95% confidence intervals [CI) was pooled for comparison between the highest compared with the lowest [reference) category of CRP level or log-transformed per unit increase in CRP level. Between-study heterogeneity was tested using the *I^2^* statistic and Cochran’s *Q* test. *I^2^* > 50% or *P*<0.10 for Cochran’s *Q* test indicate high heterogeneity. When significant heterogeneity was present, we applied a random-effect model. Otherwise, a fixed-effect model was selected. We performed the subgroup analyses by the study design [prospective or retrospective), length of follow-up [first 3 months compared with >3 months), geographic area [Europe compared with others), sampling time of the blood [within 72 h compared with 72 h to 15 days), cut-off values of CRP [single compared with ≥3 categories), measurement of CRP [high-sensitivity compared with routine), and NOS [≥7 compared with <7 points). Sensitivity analyses were performed using the leave-one-out method. Publication bias was evaluated using the Begg’s test and Egger’s test, with *P*>0.1 indicating no statistical significance.

## Results

### Search results and study characteristics

**Figure 1:**
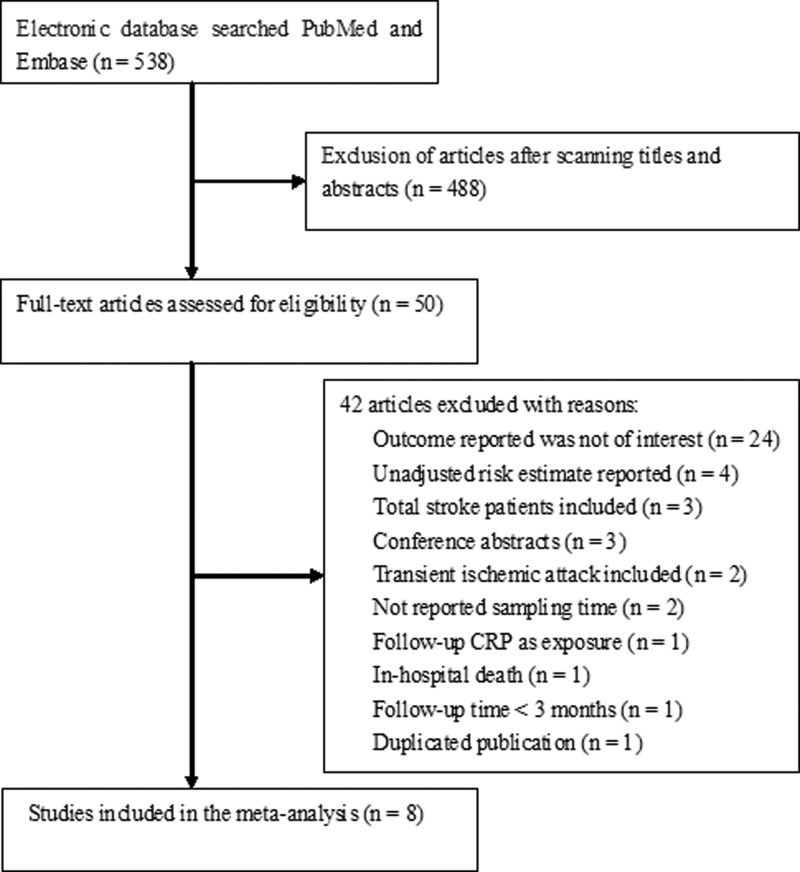
Flow diagram of the study selection process.

Of the 538 relevant records obtained from the initial electronic search, 8 studies [15,19–25] were finally included in the meta-analysis. Figure 1 lists the detailed process of the study selection. The baseline characteristics of the selected studies are summarized in Table 1. A total of 3604 acute ischemic stroke patients were identified. The sample size of the included studies ranged from 228 to 741. Five studies [19,20,23–25] were prospective and three [15,21,22] were retrospective in nature. Five studies [15,19,21–23] were carried out in Europe, two in China [24,25], and one in the United States [20]. Follow-up duration ranged from 3 months to 7.4 years. Blood samples were collected on admission or within 15 days of symptom onset. Two studies [20,24] measured CRP by means of a high-sensitivity method. The NOS score of the included studies ranged from 6 to 8 points, which could be considered as moderate to high quality.

**Table 1:** Baseline characteristics of studies included in meta-analysis

Author/year	Region	Study design	Patients (% men)	Age/range Mean (S.D.)	CRP cut-off value (mg/l)	Sampling time	Death numbers	HR/OR (95% CI)	Follow-up	Adjustment for covariates	Overall NOS
Muir et al. 1999 [19]	Scotland	Prospective study	AIS 228 (54.4)	67.0 ± 13.1	Log- transformed per unit increase	Within 72 hours of admission	81	1.23 (1.13–1.35)	2.63 years	Age, NIHSS, TC, histories of previous MI, stroke, and cigarette smoking	7
Elkind et al. 2006 [20]	U.S.A.	Prospective study	AIS 467 (45.4)	68.9 ± 12.7	Tertile 3 vs. 1; ≥31.2 vs. <4.2 (Hs-CRP)	Hospitalization or clinic visit	159	2.11 (1.18–3.75)	4.0 years	Age, sex. race/ethnicity, CAD, DM, hypertension, hyperlipidemia, AF, current smoking, stroke severity and Lp-PLA2	7
Shantikumar et al. 2009 [21]	U.K.	Retrospective study	AIS 394 (50.8)	58–81	Quintile 4 vs. 1; >22.3 vs.<2.48	Within 10 days of symptom onset	231	2.0 (1.3–3.1)	7.4 years	Age, AF, previous stroke/TIA, stroke subtype	6
den Hertog et al. 2009 [22]	The Netherlands	Retrospective study	AIS 561 (60.0)	69.7 ± 13.5	≥7.0 vs. <7.0 and log-transformed per unit increase	Within 12 h of symptom onset	110	1.7 (1.0–2.9), 1.9 (1.2–2.8)	3 months	Age, sex, NIHSS, cigarette smoking, DM, hypertension, statin use, and stroke subtype	7
Idicula et al. 2009 [23]	Norway	Prospective study	AIS 498 (60.6)	69.3 ± 14.0	Tertile 3 vs. 1; ≥10.0 vs. <3.0	Within 24 h	67	3.47 (1.58–7.64)	2.5 years	Age, sex, NIHSS, pre-existing DM and intravenous thrombolysis in categories	8
Huang et al 2012 [24]	China	Prospective study	AIS 741 (74.9)	60.9 ± 13.3	>3.0 vs. ≤3.0 (Hs-CRP)	Within 15 days of symptom onset	32	6.48 (1.41–29.8)	3 months	Age, sex, NIHSS, hypertension, CAD, and fasting glucose at admission	6
Karlinski et al. 2014 [15]	Poland	Retrospective study	AIS 341 (32.9)	55–74	>5.0 vs. ≤5.0	Within 24 h after admission	53	1.32 (0.51–3.37)	3 months	Age, baseline NIHSS, DM, CHF, lack of prestroke disability, recent infection and prestroke statins use	7
Li et al. 2015 [25]	China	Prospective cohort study	AIS 374 (55.1)	69 (63–79)	Log-transformed per unit increase (Hs-CRP)	Within 12 h of symptom onset	64	15.37 (3.25–41.08)	1 year	Age, sex, smoking, glucose, NIHSS, homocysteine, procalcitonin, infarct volume, and rt-PA-treated	7

Abbreviations: AF, atrial fibrillation; AIS, acute ischemic stroke; CAD, coronary artery disease; CHF, congestive heart failure; DM, diabetes mellitus; HR, hazard ratio; Hs-CRP, high-sensitivity CRP; Lp-PLA2, lipoprotein-associated phospholipase A2; MI, myocardial infarction; NIHSS, National Institutes of Health Stroke Scale score; OR, odds ratio; rt-PA, recombinant tissue plasminogen activator.

### Categorical analysis of CRP with all-cause mortality

**Figure 2:**
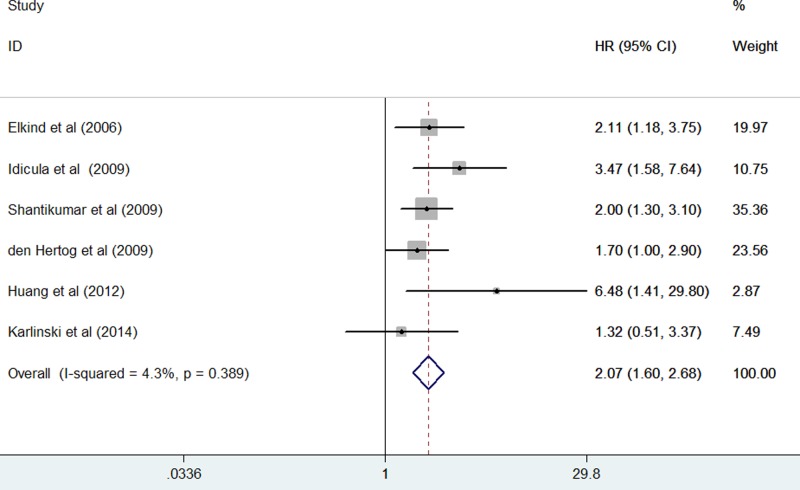
Forest plots showing RRs with 95% CIs of all-cause mortality for the highest compared with the lowest CRP level category

Six studies [15,20–24] reported the all-cause mortality risk by the categorical analysis of CRP elevation. As shown in Figure 2, no significant heterogeneity across studies was found (*I^2^* = 4.3%; *P*=0.389). Meta-analysis showed that the pooled hazard ratio (HR) of all-cause mortality for the highest compared with the lowest CRP category was 2.07 (95% CI: 1.60–2.68) in a fixed-effect model.

### Continuous analysis of CRP with all-cause mortality

**Figure 3:**
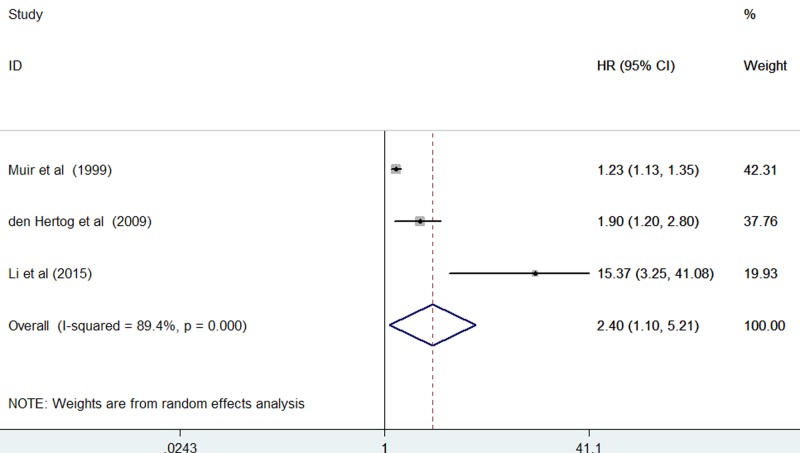
Forest plots showing RRs with 95% CIs of all-cause mortality for per unit increase in log-transformed CRP level

Three studies [19,22,25] reported the all-cause mortality risk by the continuous analysis of CRP increment. Meta-analysis showed that the pooled HR of all-cause mortality for per unit increase in log-transformed CRP was 2.40 (95% CI: 1.10–5.21) in a random-effect model (Figure 3). A leave-one-out sensitivity analysis demonstrated that removal individual study did not alter the overall pooled risk estimate in both categorical and continuous analyses (data not shown).

### Subgroup analyses and publication bias

Subgroup analyses showed that the pooled HR of all-cause mortality was 1.80 (95% CI: 1.16–2.81) and 2.26 (95% CI: 1.65–3.08) in the first 3 months and more than 3 months follow-up, respectively. In addition, statistical associations were consistently observed in each subgroup (Table 2). No evidence of significant publication bias was found according to the Begg’s test (*P*=0.452) and the Egger’s test (*P*=0.293).

**Table 2:** Subgroup analyses of CRP elevation with acute ischemic stroke risk

Subgroup	Number of studies	Pooled HR	95% CIs	Heterogeneity between studies
Study design				
Prospective	3	2.72	1.74–4.25	*P*=0.308; *I^2^* = 15.0%
Retrospective	3	1.80	1.31–2.47	*P*=0.710; *I^2^* = 0.0%
Region				
Europe	4	1.97	1.47–2.65	*P*=0.396; *I^2^* = 0.0%
Others	2	2.43	1.41–4.17	*P*=0.178; *I^2^* = 45.0%
Cut-off value of CRP				
Single	3	1.80	1.16–2.81	*P*=0.205; *I^2^* = 36.9%
Tertile/Quartile	3	2.22	1.62–3.06	*P*=0.476; *I^2^* = 0.0%
Sampling time				
Within 72 h	4	2.00	1.44–2.78	*P*=0.390; *I^2^* = 0.4%
10–15 days	2	2.18	1.44–3.32	*P*=0.146; *I^2^* = 52.6%
Measurement of CRP				
High-sensitivity	2	2.43	1.41–4.17	*P*=0.178; *I^2^* = 45.0%
Routine	4	1.97	1.47–2.65	*P*=0.396; *I^2^* = 0.0%
Follow-up duration				
First 3 months	3	1.80	1.16–2.81	*P*=0.205; *I^2^* = 36.9%
>3 months	3	2.22	1.62–3.06	*P*=0.476; *I^2^* = 0%
Study quality				
NOS ≥ 7	4	2.11	1.43–3.10	*P*=0.158; *I^2^* = 42.2%
NOS < 7	2	2.04	1.44–2.89	*P*=0.885; *I^2^* = 0.0%

## Discussion

This meta-analysis demonstrated that CRP elevation was independently associated with an increased risk of all-cause mortality in patients with acute ischemic stroke. Acute ischemic stroke patients with the highest CRP elevation had a 2.07-fold risk of all-cause mortality. Moreover, prognostic strength of CRP elevation was clearly supported by each unit increase in log-transformed CRP by 2.40-fold all-cause mortality risk. These findings raise the possibility adding CRP to the prognostic model will improve the prediction of post-stroke death events.

Accurately predicting stroke prognosis is challenging. Blood biomarkers may provide useful information to improve the prediction of outcome after ischemic stroke [26]. Stroke prognosis can be classified by survival, neurological, and functional outcomes. The prognostic value of CRP in patients with acute ischemic stroke has been widely investigated. Elevated baseline higher CRP level could predict an unfavorable long-term functional outcome in patients with ischemic stroke [16]. However, the role of CRP in predicting the survival outcome amongst ischemic stroke remains controversial.

Subgroup analysis showed a stronger all-cause mortality risk in the studies with more than 3 months of follow-up than in the first 3 months. This finding may be partly explained by the highest risk of recurrent stroke and sudden cardiac death during the early period post stroke. CRP elevation can increase the risk of recurrent stroke [27,28], which was associated with an increased risk of death [29]. Sudden cardiac death may be the leading cause of post-stroke mortality because of the stronger association between CRP and cardiac injury [30,31]. Furthermore, exclusion of patients with an early infection during hospitalization did not significantly affect the association of CRP with mortality [22]. Thus, the predictive value of CRP in acute stroke appeared to be altered by early infection. Therefore, screening CRP level can improve the prediction of both long-term and short-time death events.

An important issue that has not been explored in the reports is the timing of blood sampling. Blood samples were collected from within 12 h to 15 days after the symptom onset or admission in the analyzed studies. However, our stratified analysis indicated that the pooled HR was similar in within 72 h and beyond 72 h time points of blood sampling subgroups. In-line with our results, CRP elevation within 30 days of stroke also highly predicted long-term survival [27]. Nevertheless, follow-up CRP elevation was an independent predictor of mortality after 90 days in patients with thrombolytic acute stroke [32]. Thus, using CRP level in predicting mortality regardless of the timing of blood sampling may be valid.

CRP elevation may reflect a systemic inflammatory response following ischemic stroke, the degree of stroke severity, or concurrent infections at the time of sampling. Stroke severity judged by the National Institutes of Health Stroke Scale score is strongly associated with mortality after ischemic stroke [33]. However, an elevated CRP level predicted future mortality independent of stroke severity. CRP elevation may also reflect a pre-existing atherosclerotic plaque or the presence of vascular risk factors. In addition, association of CRP with post-stroke mortality may partly reflect inflammation-induced endothelial cell dysfunction and platelet activation [21]. All these factors contributed to the excessive risk of post-stroke mortality.

This meta-analysis has several limitations. First, single CRP determination at baseline rather than several measures may have led to the misclassification of patients in each category. Second, an elevated CRP level may reflect a state of concurrent autoimmune diseases or chronic inflammatory disease during the study enrolment. Lack of adjustment for these conditions may have overestimated the prognostic significance of CRP. Third, various cut-off values of CRP elevation have been reported in the selected studies, and but we could not determine an optimal threshold of the CRP. Fourth, all the patients were diagnosed with acute ischemic stroke, so the current findings could not be generalized to transient ischemic attack and hemorrhagic stroke. Furthermore, the prognostic role of CRP may also be affected by different phases or subtypes of ischemic stroke. Finally, elevated CRP level after thrombolysis treatment did not independently predict the outcome in patients with ischemic stroke, suggesting that tissue plasminogen activator may influence the prognostic significance of CRP [15]. Future studies of blood samples performed directly before the tissue plasminogen activator treatment are required.

In conclusion, patients with acute ischemic stroke that have elevated CRP levels have a significantly increased risk of all-cause mortality. CRP level measurement should be considered for post-stroke death risk stratification in patients with acute ischemic stroke. Future well-designed prospective studies should be conducted to assess the prognostic values of other inflammatory biomarkers or concurrently multiple markers in ischemic stroke patients.

## Abbreviations

CI: confidence interval
CRP: C-reactive protein
HR: hazard ratio
NOS: Newcastle–Ottawa Scale
